# From Molecular Simulations to Experiments: The Recent Development of Room Temperature Ionic Liquid-Based Electrolytes in Electric Double-Layer Capacitors

**DOI:** 10.3390/molecules29061246

**Published:** 2024-03-11

**Authors:** Kun Zhang, Chunlei Wei, Menglian Zheng, Jingyun Huang, Guohui Zhou

**Affiliations:** 1 Institute of Wenzhou, Zhejiang University, Wenzhou 325036, China; 2 Institute of Thermal Science and Power Systems, Zhejiang University, Hangzhou 310027, China; 3 Institute of Quzhou, Zhejiang University, Quzhou 324000, China; 4 State Key Laboratory of Silicon Material, Zhejiang University, Hangzhou 310027, China; 5 School of Chemistry and Chemical Engineering, Qingdao University, Qingdao 266071, China

**Keywords:** room temperature ionic liquid-based electrolyte, molecular dynamics simulation, electric double-layer capacitors

## Abstract

Due to the unique properties of room temperature ionic liquids (RTILs), most researchers’ interest in RTIL-based electrolytes in electric double-layer capacitors (EDLCs) stems from molecular simulations, which are different from experimental scientific research fields. The knowledge of RTIL-based electrolytes in EDLCs began with a supposition obtained from the results of molecular simulations of molten salts. Furthermore, experiments and simulations were promoted and developed rapidly on this topic. In some instances, the achievements of molecular simulations are ahead of even those obtained from experiments in quantity and quality. Molecular simulations offer more information on the impacts of overscreening, quasicrowding, crowding, and underscreening for RTIL-based electrolytes than experimental studies, which can be helpful in understanding the mechanisms of EDLCs. With the advancement of experimental technology, these effects have been verified by experiments. The simulation prediction of the capacitance curve was in good agreement with the experiment for pure RTILs. For complex systems, such as RTIL–solvent mixtures and RTIL mixture systems, both molecular simulations and experiments have reported that the change in capacitance curves is not monotonous with RTIL concentrations. In addition, there are some phenomena that are difficult to explain in experiments and can be well explained through molecular simulations. Finally, experiments and molecular simulations have maintained synchronous developments in recent years, and this paper discusses their relationship and reflects on their application.

## 1. Introduction

With the application of energy storage devices becoming increasingly extensive [1,2,3,4,5,6,7], research on their related electrode materials [8,9,10] and electrolytes has become more widespread [11] and in-depth [12,13,14,15]. Electric double-layer (EDL) capacitors (EDLCs) are capable of quick charge/discharge rates and large charge storage densities; their process of charge/discharge has gained attention in the electrochemical research field. They are nonfaradaic energy storage devices. They occupy a middle ground between batteries and dielectric capacitors, and are adaptable enough to be used as standalone energy storage devices or in conjunction with batteries. EDLCs are appropriate for situations that need reliability and quick charge/discharge process, such as uninterruptible power systems, high-performance filter capacitors, energy recovery devices, and buffers for the power grid. This is because they outperform dielectric capacitors and batteries in terms of energy density, cycle life, and power density.

The majority of commercial EDLCs use organic electrolytes over a long time; this is because they are volatile and combustible despite having a larger working voltage window (over 2.5 V) when compared to aqueous electrolytes (1.23 V operating limit). However, room temperature ionic liquids (RTILs) have received much interest because they perform better in terms of EDLC electrolytes than common organic electrolytes; the electrochemical window of RTILs often reached 3–4 V or even 7 V [16]. On the other hand, RTIL-based EDLCs can be adapted to a wide range of environmental conditions. This is because RTILs [17] are salts composed of an organic cation and either organic or inorganic anions that can avoid the formation of organized crystalline structures. Furthermore, their liquid temperature range is relatively wide, ranging from negative over 100 to positive hundreds of degree.

Therefore, more and more researchers are becoming concerned about the energy storage mechanisms for RTIL-based EDLCs. To take a closer look, the EDL has been emerging as a focal point of RTIL-based EDLCs, owing to its fundamental importance in electrochemical energy storage and chemical conversions. However, although it is universally acknowledged that the structure of EDL determines the interface, it is rarely investigated. This is likely due to the difficulty associated with characterization. Even advanced characterization techniques, such as scanning probe microscopy (SPM) [18], have been utilized. The dynamic evolution of the EDL response to applied bias potentials makes it difficult to accurately capture the evolving charge properties and molecular-scale behavior using current techniques in a non-vacuum environment [19]. Therefore, the practical phenomena can only be explained by infinitely modified model theories, meaning scientists are increasingly investigating these simulations.

Researchers have made many attempts to provide a reasonable description of the EDL behavior of RTILs. Kilic et al. [20,21] discovered that the classic Gouy–Chapman theory was more suited for dilute electrolytes that did not depend on ionic volume, as opposed to describing the electrolyte behavior for RTILs.

Simulations, which can offer more information and are simpler to manipulate when compared to experiments, can be helpful to obtain a better understanding of the charge storage mechanism. From the viewpoint of energy changes, several discoveries have been obtained via simulations. This method has recently been one of the most effective techniques for analyzing physical and chemical processes, including the finite element method (FEM), density functional theory (DFT), and molecular simulation, which have been used to describe the capacitive properties of EDLCs. Due to the high computational costs, it is difficult to expand DFT simulations to a realistic description of complicated ion structures proximate to porous electrodes. Furthermore, the capacitive behaviors of porous electrodes with concentrated electrolytes (e.g., RTILs) are frequently missed by FEM calculations, which are typically characterized using a continuum model (e.g., Nernst–Plank equations).

Molecular simulations allow users to manipulate the system specifics and fundamental temporal dynamics. The amount of time required to create computer files and scripts is significantly decreased using a commercial tool. In theory, atomic-level details of the structures and movements can be provided using molecular simulations. However, due to the absence of precise reactive force field potentials, it is still difficult for molecular simulations to accurately depict the reaction or conversion process of pseudocapacitance. An advantage of simulations is the ability to forecast the physicochemical phenomena on EDLCs. The Monte Carlo (MC) method and molecular dynamic (MD) simulations are two examples of molecular simulations.

MD simulations are a useful modeling technique for describing particle interactions and simulating their dynamic behaviors within nanoscale physical events. By using numerical methods, such as the Verlet technique, to solve Newton’s equations of motion, the MD system can characterize the physical movements of atoms and molecules. The MD system can also be used to elucidate the interatomic potentials or force fields of the forces that are applied to the particles. Comprehensive information regarding the particles’ movements and trajectories throughout the whole process is revealed through iterations between determining the motion of the particles and computing the force. MD simulations can provide a complete image of these dynamics by following the paths of the particles in order to obtain the macroscopic properties of the simulation system.

MC simulations are simpler than MD simulations. This technique simulates mathematical issues and employs random numbers to create solutions that might not be completely random. The forces are also not required for the computation. The potential energy function is generated for each of the many configurations (geometries) that make up an MC simulation, which is a stochastic simulation. Then, the thermodynamic characteristics are computed using these data. The MC approach computes the time-averaged characteristics of a many-body system via solving Newton’s equation of motion, which is a simple concept.

Thanks to molecular simulations, valuable insights into the atomic and electronic structure of the EDL can be provided, allowing a full understanding of the interface. The reconstruction of the EDL structure during electrodeposition is visualized under the potential of zero charge and different applied voltages. When combined with the corresponding interfacial cumulated density profiles of the species, the orientational rearrangement of ions from RTILs and the dynamic evolution of ions and solvated ions are illustrated. Moreover, researchers use molecular simulations to investigate the effect of RTIL conformational transitions on charge/discharge processes, providing details not observed under standard experiment measures. Therefore, using experimental means alone, one cannot quantitively describe interfacial conformations. The molecular simulations can not only save a lot of resources, but can obtain some important local data that cannot be gathered in experiments. Minor changes of nano-structures will significantly change EDL properties, and even current experimental techniques can not accurately measure the structures of ions in EDLs. It can be said, therefore, that molecular simulations gain their domination in advancing this research field, often outperforming using experimental means alone.

In the past, some researchers reviewed this field from different perspectives. Some provided macroscopic views for experimental and engineering applications [22], while others broadly discussed various kinds of simulation methods [16,23,24,25]. Certain reviews focus predominantly on experiments [25,26,27], whereas others were more concerned with EDLs of barriers [28] or pseudocapacitance [29] relating to RTIL-based electrolytes.

Here, this analysis combines molecular simulations and experimental comparisons to investigate the dynamics of various effects influencing RTIL-based EDLCs.

## 2. Supposition from Molecular Simulations and Verification from Experiments

To determine the distributions and motions of electrolyte ions in EDLCs, one may first define ion routes and interactions, which represent their dynamic behavior and energy storage ability, respectively. Due to their fundamental nature, molecular simulations advance our understanding and aid in improving the capabilities of EDLCs made from RTILs via offering atomic level-insights into the mechanics of charge storage and ion transport. A precise way to look at the difficulties and complex phenomena that exist in these systems is through using molecular simulations.

**Figure 1:**
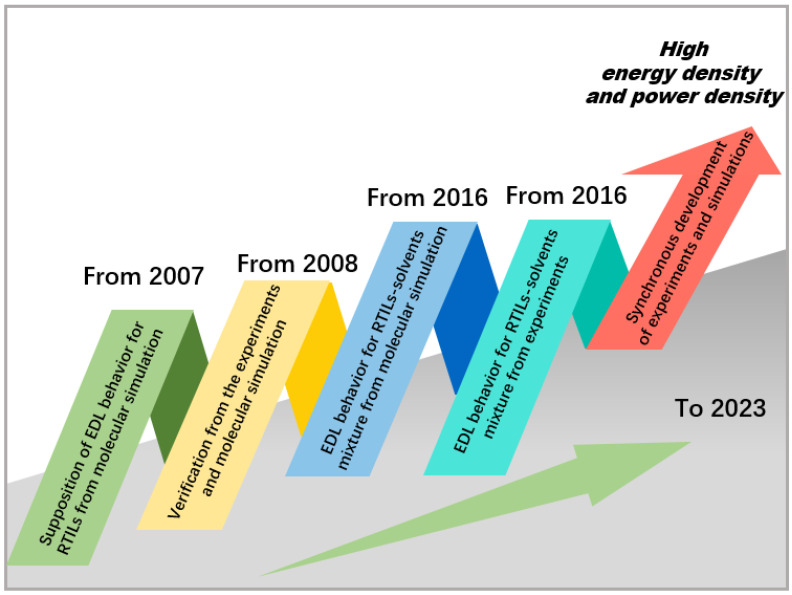
The timeline for the molecular simulations and experiments of RTIL-based electrolytes in EDLCs.

In 2007, due to the lack of theories and simulations regarding potentials of zero charge (PZC) in EDCL capabilities for RTIL-based electrolytes [21], the experimental results were still scarce and to a large extent inconclusive. Alexei A. Kornyshev et al. studied the MC simulations from Booth and Haymet, which can be used as a reference for RTIL-based electrolytes; this concerns the comparison of the “singlet”-integral equation theory of molten salts [30]. Based on this, and assisted by other theoretical works, Alexei A. Kornyshev et al. [21] stated that the capacitance curves in RTILs are far from being Gouy–Chapman-like curves. Experiments in 2008 revealed that the differential capacitance/potential curve has a minimum point and two side branches [31]. Additionally, in the same year, MD simulations also demonstrated the behavior of Cd concerning electrode potential. This behavior manifested as either a bell-shaped (single hump) or camel-shaped (double hump) curve [32], indicating that the relationship between Cd and U near the PZC depends on the compatibility of the RTILs. These findings contrasted with the U-shaped curve derived from the Gouy–Chapman theory (in fact, a curve that was more camel-shaped was shown by MC simulations up until 2010 [33,34]). Through experiments and molecular simulation studies, we can better understand this phenomenon and provide guidance for the design and application of RTIL electrochemical systems. The process was shown in Figure 1.

**Figure 2:**
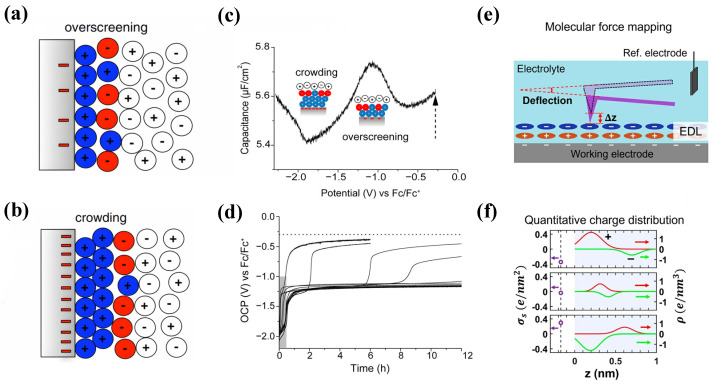
Experimental studies on overscreening and crowding effects for RTIL-based electrolytes. (**a**,**b**) Schematic of overscreening and crowding effects [35]. Copyright2011, American Physical Society. (**c**,**d**) Experimental evidence of overscreening and crowding effects from open circuit potential (OCP) measurements [36]. Copyright2021, American Chemical Society. (**e**,**f**) Experimental evidence of the overscreening effect from 3DAFM measurements [37]. Copyright2022, American Chemical Society.

Molecular simulations play a critical role in advancing the field of EDLCs and accelerating their adoption across various applications. Therefore, in the early research work focusing on RTIL bilayers, the progress of molecular simulations was generally faster than that of experiments. For example, in 2010, Bazant et al. [35] first used MD simulations to discover that, under low voltages, the RTILs would undergo an overscreening effect near the electrode, as shown in Figure 2a. Under higher voltages, the crowding of counterions becomes easier, as shown in Figure 2b.

Subsequently, experiments were conducted to verify the two predicted phenomena. However, there are still some questions regarding the crowding and overscreening effect in the experiments. Mattia Belotti et al. [36] detected and verified the two effects of [C_6_mim]^+^, [C_4_mim]^+^, and [C_2_mim]^+^ using a simple open circuit potential method, as shown in Figure 2c,d. However, they failed to verify the alternating changes of these two effects for ions such as [TDTHP]^+^, [pyr_14_]^+^, [PF_6_]^−^, and [EtSO_4_]^−^.

Chu et al. [38,39] conducted experiments on [TDTHP][NTf_2_], [N_4,1,1,1_][NTf_2_], and [N_1,8,8,8_][NTf_2_] systems using in situ synchrotron radiation X-ray reflection. They were able to directly confirm the theoretical prediction of ion crowding near the interface. For [TDTHP]^+^, [N_4,1,1,1_]^+^, and [N_1,8,8,8_]^+^ under a negative voltage, no crowding or overscreening effects were observed. This was determined by characteristic reflectance curves. Chu et al. also discovered that the congestion effect in the [NTf_2_]^−^ crowding experiment occurs at a low threshold voltage of V_th_ ≈ 1.75 V, which contradicts previous simulations, which had predicted that crowding would occur at a higher voltage. They also tested the [TDTHP][NTf_2_] system, which has the same cation as [TDTHP][NTf_2_], but has a different anion, and they did not observe any related EDL phenomena. In contrast, Kornyshev et al. [40] found that only extremely exaggerated surface charge density values (σ = −120 to +120 µC/cm^2^) were able to simulate the crowding behavior on the electrode surface. They used the latest results from MD simulations in 2020 to investigate crowding behavior during the charging process. This indicates that their ideal hard sphere model still has some discrepancies with experimental observations.

Recently, according to Narayana R. Aluru et al. [41], the molecular size EDL architectures of RTILs were directly imaged using an advanced electrochemical three-dimensional atomic force microscope (EC-3DAFM) at various electrode potentials. The oscillation and width characteristics of the first layer of the EDL are significantly reconstructed with changes made in the electrode potential, while the changes in other layers are relatively weak. Based on experimental results and the MD simulation, the electrode potential, which is essential for capacitive charge storage, substantially influences the spatial organization and tilt angle of cations and anions in the first layer of the EDL. Two years later [37], they further developed the charge profiling three-dimensional atomic force microscope (CP-3D-AFM) for charge distribution, which could observe Angstrom resolution observations of the true spatial charge distribution near the electrodes. It was found that the integrated charge density was consistent with the macroscopic electrochemical measurement results. Their results further explore the overscreening effect and are a good example of the combination of experiments and molecular simulation techniques, as shown in Figure 2e,f.

## 3. Studies on the Mechanism of the Charging and Discharging Process

One advantage of using molecular simulations is observing the movement processes of nanoscale molecules. Therefore, using molecular simulations to observe the charging and discharging processes at the interface between RTIL-based electrolytes and electrodes has received intense attention. The research process in this field has two characteristics. First, after the prediction of molecular simulations at the nanoscale, experiments are conducted to verify and correct them. This process generally requires experimental conditions to be sufficiently mature in order to successfully validate the results from simulations. A detailed introduction has already been provided in the previous section. Second, based on the latest experimental results, molecular simulation methods are used to analyze and explain their mechanisms. This process is more common and fully leverages the advantages of molecular simulations.

### 3.1. Studies on the Mechanism of the Charging Process

**Figure 3:**
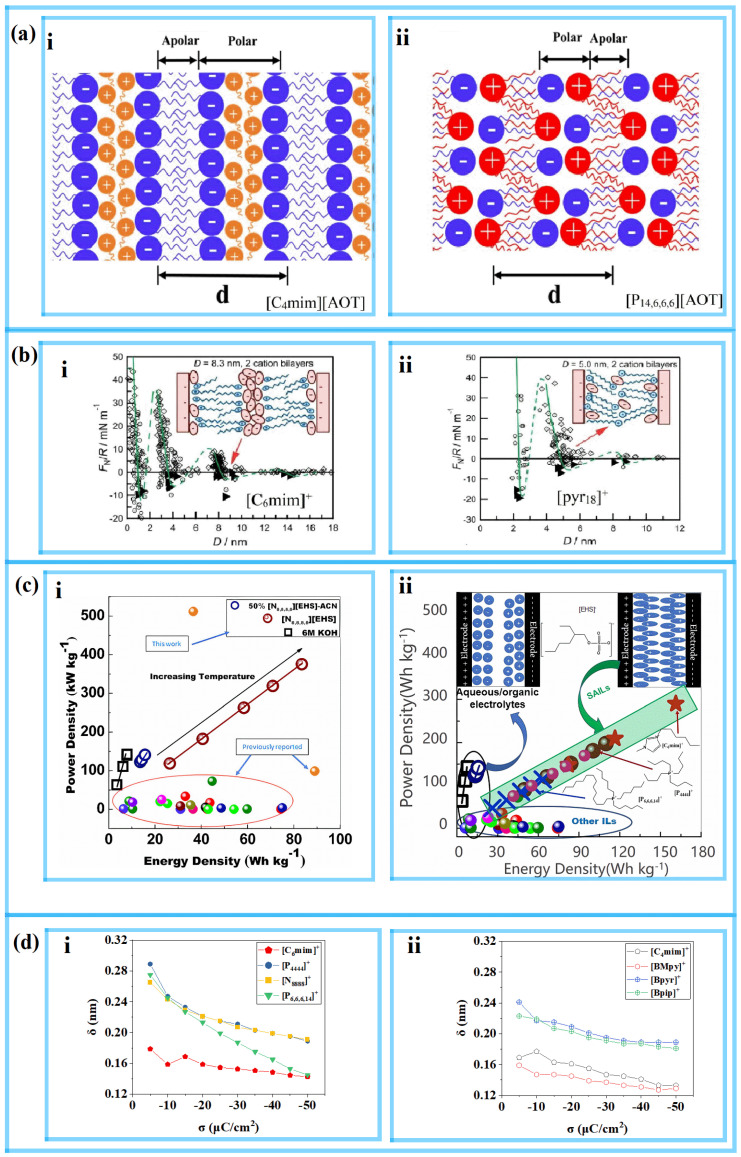
The EDL structure for the self-assembly behavior of the RTIL experiments and MD simulations during charging. (**a**) Schematic diagram of the difference in self-assembly behaviors between imidazolium-based SAILs and catanionic SAILs [48]. Copyright2022, Elsevier. (**b**) Schematic illustration showing how pyrrole and imidazole cations behave differently during self-assembly [49,50]. Copyright2013, Royal Society of Chemistry, American Chemical Society. (**c**) Ragone plots for a supercapacitor cell with different kinds of SAILs [47,51]. Copyright2021, American Chemical Society; Copyright2022, Elsevier. (**d**) The distance between the electrode surface and the peak of the cation’s *ρ*_n_ curve [53]. Copyright2023, Elsevier. (**i**) Systems with the anion [C_8_SO_4_]^−^. (**ii**) Systems with the anion [AOT]^−^.

**Figure 4:**
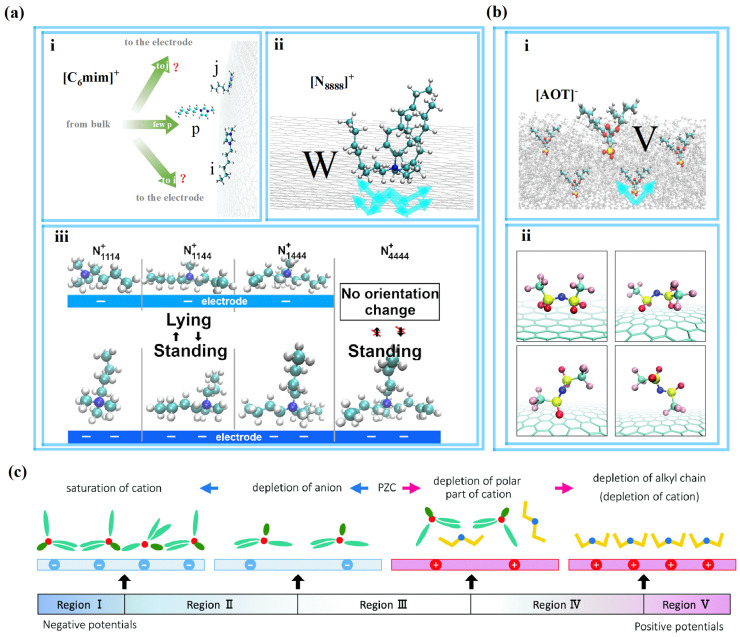
The conformation of ions near the electrodes during the charging process. (**a**) The cations close to the negative electrode. (**i**) The imidazolium-based cations. (**ii**) Surfactant-like cations [53]. Copyright2023, Elsevier. (**iii**) Schematic illustration of [N_1,1,1,4_]^+^, [N_1,1,4,4_]^+^, [N_1,4,4,4_]^+^, and [N_4,4,4,4_]^+^ in the first ionic layer viewed from the lateral direction [45]. Copyright2020, American Chemical Society. (**b**) The anions near the positive electrode. (**i**) Surfactant-like anions [43]. Copyright2021, American Chemical Society. (**ii**) Snapshots of [NTf_2_]^−^ in the first ion layer [46]. Copyright2020, Royal Society of Chemistry. (**c**) Schematic illustration of the behavior of [N_1,4,4,4_][NTf_2_] at the EDL [46]. Copyright2020, Royal Society of Chemistry.

Researchers often compare the energy storage performance of different types of RTIL-based electrolytes, and the atomic structure of their nanoenvironment at the interface near the electrode is also receiving increasing attention. Surface-active ILs (SAILs) have attracted the interest of many researchers. Initially, SAILs were studied for their self-assembly processes at the nanoscale level, and were recently discovered to have good potential as electrolytes for EDLCs. Studies have found that SAIL-based electrolytes have both hydrophilic and hydrophobic carbon chains, meaning that they exhibit excellent energy storage performance even at high temperatures, which is a characteristic that nonamphiphilic ILs (NAILs) do not have. Recently, Mao et al. [42] discovered nonpolar regions formed by tail-to-tail connections in imidazolium-based SAILs (such as [C_4_mim][AOT]) near the electrode, as shown in Figure 3a(i). These regions can suppress the occurrence of overscreening effects and provide better performance in electrochemical storage than NAILs, particularly at high temperatures. Zhou et al. [43] used MD simulations to reveal the structural transition of SAILs under a rapid charge/discharge process. Near the positive electrode of the [C_4_mim][AOT] system, it was found through the use of MD simulations that [AOT]– easily entered the interfacial layer with a V-shaped conformation during the charging process, as shown in Figure 4b(i). After a period of charging and discharging, the electrolyte included more V-shaped anions than in the simulated beginning condition, which how the electrolyte was optimized. This can be likened to the conformational change of butyl chains from lying down to standing up, as reported by Sakka et al. [44,45,46]. This change occurs in quaternary ammonium ILs, such as [N_1,1,4,4_][NTf_2_], [N_1,4,4,4_][NTf_2_], [N_1,1,4,4_][NTf_2_], and [N_4,4,4,4_][NTf_2_], as shown in Figure 4a. It is proposed that in addition to the ion densification in the first ion layer, the model also needs to consider the orientation change of the ions during the charging process.

The electrolyte systems of [TDTHP][AOT] and [N_8,8,8,8_][EHS] have also received attention in recent experimental studies. Although they are also classified as SAILs, they differ from [C_4_mim][AOT], which has an imidazolium-based cation, in that their cations are surfactant-like ions. Therefore, these are referred to as catanionic SAILs. With superior energy storage capabilities compared to NAILs, both imidazolium-based and catanionic SAILs can be employed as electrolytes or electrolyte components in supercapacitors and batteries over a wide temperature range. As shown in Figure 3c(i), Jain et al. [47] revealed that [N_8,8,8,8_][EHS] works better than NAILs at high temperatures. According to Zhang et al.’s [48] structural observation, [TDTHP][AOT] exhibits a sponge-like nanostructure with a clear cation-anion alkyl chain cross, as seen in Figure 3a(ii). Imidazolium-based SAILs have a stronger interfacial layering structure than [TDTHP][AOT]. Because both its cation and anion are surfactant ions, catanionic SAILs differ from [C_4_mim][AOT] in terms of the self-assembly of the nanostructure. The alkyl chains from both the [TDTHP]^+^ (trihexyltetradecylphosphonium, as shown in Figure 3c(ii), also named [P_6,6,6,14_]^+^) cation and [AOT]^−^ anion tend to cross within the double-ion layer, resulting in a shorter distance between the polar and nonpolar structural layers.

In earlier experimental studies, Perkins et al. [49,50] reported variations due to the different alkyl chain lengths of the [C*_n_*mim]^+^ cations of imidazolium-based RTILs. Cations with shorter alkyl chain (*n* ≤ 4) substituents form alternating cation-anion monolayers in the space confined by the charged plane, while cations with longer alkyl chain substituents exhibit a “toe-to-toe” self-assembly structure due to the amphiphilicity of ions. This phenomenon is particularly evident when *n* = 6, as shown in Figure 3b(i). However, when *n* = 10, they found that the “toe-to-toe” bilayer structure of imidazolium-based RTILs no longer appeared. Perkins et al. [49,50] also found that for pyrrole RTILs with [pyr_1n_]^+^, “cross” self-assembled structures occur between chain lengths of *n* = 8 and 10, and chain lengths of *n* < 8 are monolayer alternating structures, as shown in Figure 3b(ii). These results indicate that the self-assembly behavior varies with the length of the alkyl chain and the different structures of the charged head.

The behavior of catanionic SAILs is similar to the “cross” self-assembly described above, where nonpolar alkyl chains from cations and anions cross in the bilayer [48], resulting in a shorter anion/cation interlayer spacing. In contrast, SAILs with imidazolium-based cations have a longer anion/cation interlayer spacing because the tail-to-tail apolar region is derived only from anions, allowing them to exhibit “toe-to-toe” self-assembly [42] with a wider neutral region. Antzutkin et al. [51] found that the specific capacitance and energy density of [C_6_mim][EHS] are both larger than those of catanionic SAILs in the temperature range of 253~373 K, such as [P_4,4,4,4_][EHS], [N_8,8,8,8_][EHS] and [TDTHP][EHS], as shown in Figure 3c(ii). Vatamanu et al. [52] reported that the differential capacitance of [C_4_mim]^+^ is 20% higher than that of [Bpy]^+^ when paired with the same anion to form an electrolyte.

The mechanism of these results has been studied using MD simulations. As seen in Figure 3d, Liu et al. [53] demonstrated that the cation containing unsaturated heterocycles had a strong propensity to align itself parallel to the electrode plate. As illustrated in Figure 4a(ii), this conformation enhances the possibility of attaining a higher energy density than other saturated heterocyclic SAILs or catanionic SAILs that feature W-shaped cations adjacent to the negative electrode.

The alkyl group of [C_4_mim]^+^ in the first ionic layer, however, leaves excess space for cations in the second ionic layer, but not for anions, thus leading to an uneven distribution of the ionic multilayer for the ionic layer structure around the negatively charged electrode. This is a finding from Tetsuo Sakka et al. [54] when using MD simulations. During this research, the team thought there were also other alkyl groups of [C_4_mim]^+^ that tended to “stand up” in the second ionic layer. This situation is neither like the overscreening effect nor the crowding effect; it may present a more complicated phenomenon for the RTILs with long alkyl chains.

Additionally, researchers conducted a deeper exploration of this complicated phenomenon. Liu et al. [53] studied [C*_n_*mim]^+^ (*n* = 4, 6, 8, 10, 12) and found a “quasicrowding” process for imidazolium-based cations. The majority of imidazolium rings adopt a parallel orientation to the negative electrode plane, representing the ordered configuration for cations. These reside in the EDL near the electrode surface, forming shapes resembling i and j, as shown in Figure 4a(i). The p-shaped cations will move to the EDL, and they are temporarily not in the EDL at the beginning of the charging process. In the next step, p-shaped cations transform into i-shaped cations, and then i-shaped cations stand their alkyl chains to form j-shaped cations. This process provides convenience for more imidazolium rings to lie in the compact layer near the electrode when they in the j shape due to the alkyl chains “stand up” and making more room for other imidazolium rings. This is a “quasicrowding” process for imidazolium-based cations that have longer alkyl chains (except [C_2_mim]^+^) during charging.

In addition, Tetsuo Sakka et al. [54] showed that after the charging process reached a particular level, as shown in Figure 4c, the two CF_3_ groups of [NTf_2_]^−^ are back against the surface of the electrode, and the four oxygen atoms and the two sulfur atoms became “legs”, supporting an anion “standing” on the surface. Furthermore, the ionic layer structure is thus capable of changing from the effect of overscreening to crowding through a monolayer for [NTf_2_]^−^ when the charge of the first ionic layer offsets the charge of the electrode surface. Most anions near the electrode have the first shape in Figure 4b(ii).

**Figure 5:**
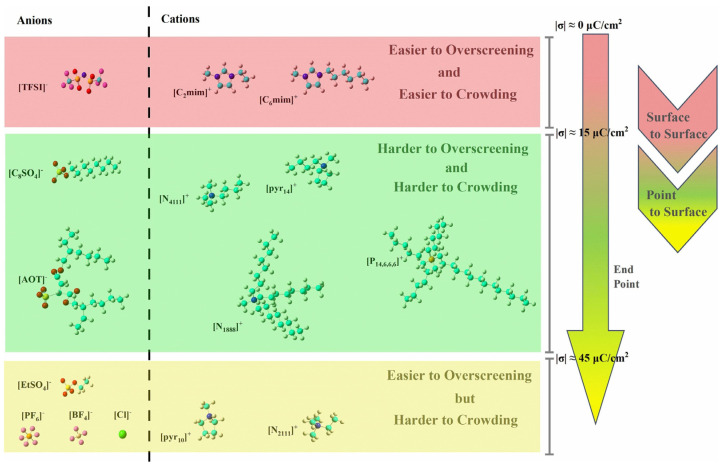
Ions of RTILs classified by their overscreening and crowding effects during the charging process [55]. Copyright2023, Elsevier.

Therefore, the nonpolar chains in ions of RTILs near the electrodes will usually “stand up” during the charging process, as shown in Figure 4. This aspect deserves close attention because these conformational changes may help to elucidate the mechanization in more detail than experiments can, as well as potentially helping to better explain the energy storage performance of RTIL-based electrolytes. Additionally, the lengths of the nonpolar chains can affect endpoints of the overscreening effect for the ions of RTIL-based electrolytes [55]. The ions of RTILs can be classified by their overscreening and crowding effects during the charging process, as shown in Figure 5, where “easier” or “harder” are relative, rather than absolute. Ions with the same charged head group and different tail lengths can be compared for different endpoints of the overscreening effect. Ions with differently charged head groups and different tail lengths are incomparable because of the number of variables involved.

**Figure 6:**
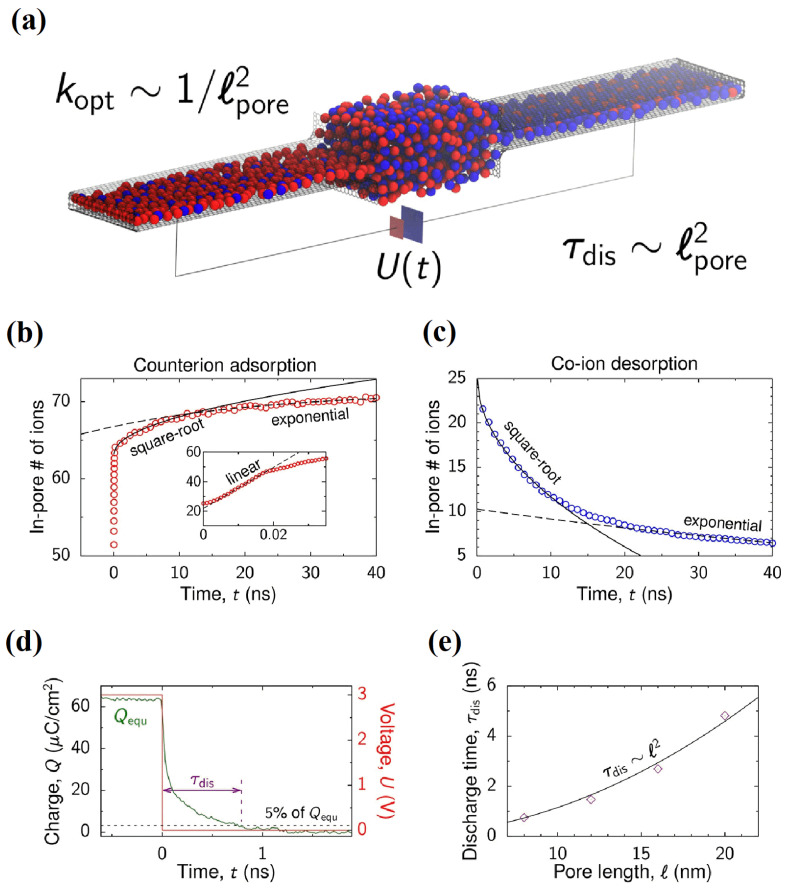
MD simulation of the charging and discharging process of a confined nanopore. (**a**) Model of a supercapacitor for molecular dynamics simulations [56]. Copyright2018, American Chemical Society. The dynamic evolution of the electrolyte in the nanoporous channels can be divided into three stages as follows: linear growth zone, square root growth zone, and exponential growth zone for counterion adsorption in (**b**) and co-ion desorption in (**c**). (**d**) Accumulated charge Q versus time for a *l* = 8 nm long pore during discharging. (**e**) Discharging time τ_dis_ as a function of pore length *l*; τ_dis_ scales as *l*^2^.

Another important application of MD simulations is to study the asymmetric dynamics of supercapacitors. As shown in Figure 6a, Kondrat et al. [56] established an MD simulation of the charging process of filling confined nanopores with RTILs, studying the charge and discharge behavior of nanoporous supercapacitors with constant interface potentials. According to the kinetic behavior of the diffusion and adsorption of electrolyte ions in nanopores, the transient formation of the RTIL phase in confined pores can lead to slow ion capture and any corresponding slow charging. As shown in Figure 6b,c, during the charging process of a supercapacitor, the charge in the nanopore gradually increases with time, and the evolution behavior of its counterions has three stages as follows: linear growth zone, square root growth zone, and exponential growth zone. However, for the co-ion, there are only the latter two evolution behaviors. It has been indicated that, if the voltage scanning rate is properly selected, the entire charging process can be significantly accelerated. At the same time, the author found that when the control voltage decreases in an appropriate range, the optimal discharge rate can be obtained.

### 3.2. Studies on the Mechanism of the Discharging Process

The discharge of energy storage equipment can typically be classified into several of the following forms: fast discharge, slow discharge, and self-discharge. Fast discharge is commonly seen in aircraft startup, camera flashes, and other devices that require instantaneous explosive power; slow discharge is relatively gentle and can be used for process such as the daily driving of zero emission electric vehicles; and self-discharge is slower and is an energy leakage during the daily storage process of energy storage equipment. Both fast and slow discharges can affect the performance of EDLCs. The self-discharge phenomenon in supercapacitors severely limits their capacity, as well as other performances.

**Figure 7:**
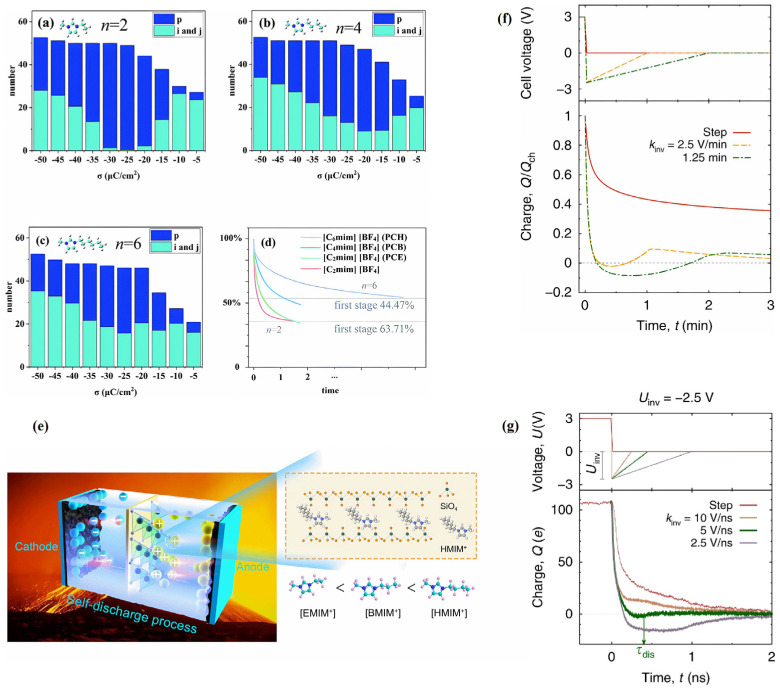
Comparison between the MD simulations and experimental data for the discharging process of the [C*_n_*mim][BF_4_] (*n* = 2, 4, 6) system. The i- and j-shaped cations are ordered; p-shaped cations are disordered [57]. Copyright2022, Royal Society of Chemistry. (**a**) represents the total number of p-, i-, and j-shaped cations with *n* = 2, (**b**) *n* = 4, and (**c**) *n* = 6. The prefix “PC” stands for poly(ethylene oxide) (PEO) and bentonite clay combinations, while the letters “H”/”B” and “E” stand for RTILs. According to Yang’s experimental findings [58], Copyright2021, Wiley. (**d**,**e**) are both schematic diagrams of the self-discharging process. The fast discharging process for [C_2_mim][BF_4_] by experiment is shown in (**f**) and by MD simulation in (**g**) [59]. Copyright2020, Springer Nature.

As mentioned above, through the MD simulation for RTIL electrolytes, Svyatoslav Kondrat et al. [56] concluded that the optimal discharge rate can be obtained when the control voltage decreases to an appropriate range, as shown in Figure 7d,e. Following this, they verified the simulation conclusion through further experiments and MD simulations [57], as shown in Figure 7f,g. Yang et al. [58] reported that when used in supercapacitors at a high temperature of 70 °C, the self-discharge during the same long time is related to the length of the alkyl chain (i.e., [C_2_mim]^+^ < [C_4_mim]^+^ < [C_6_mim]^+^). They predicted that the primary cause of the aforementioned behavior may be the induction effect of electron repulsion. The induction effect decreases with increasing alkyl chain lengths. Therefore, the adsorption capacity of [C_6_mim]^+^ on bentonite clay is much greater than that of [C_4_mim]^+^ and [C_2_mim]^+^. However, Mattia Belotti et al. [36] thought that the reason was more likely to be the projected dipole moment of the cations and the strong hydrogen bonds in the electric field, rather than the materials capable of adsorbing cations. This explanation is more reasonable because most [C*_n_*mim]^+^ ions are more strongly influenced by the electrode at the start of the discharge than when they come into contact with the bentonite clay in center of the electrolyte. The tiny variations between the red and green lines in Figure 7d show that the inclusion of poly(ethylene oxide) (PEO) and bentonite clay into identical RTILs ([C_2_mim][BF_4_]) only slightly altered the results. The green, blue, and violet line trends for cations with various chain elongations, however, demonstrate more notable differences in the first stage of discharge. Therefore, the explanation for this problem should focus on the changes in the EDL near the electrode.

The mechanism of these results was also studied using MD simulations. Near the negative electrode of [C*_n_*mim][AOT] and [C*_n_*mim][BF_4_] systems, the structural transition of imidazolium-based NAILs and SAILs in a two-stage discharge process was discussed by Zhou et al. [57]. The two-stage discharge process of NAILs was more obvious, and this process was slower for SAILs. It was uncovered that the rapid decrease in electrical energy during the first stage of discharge was primarily attributed to changes in cation shapes rather than their movement. Conversely, the gradual decrease in the second stage was induced by the overscreening effect and the slow movement of cations away from the negative electrode. Unlike the conventional view, it was concluded that the large-scale movement of ions plays a relatively minor role in releasing electrical energy.

Therefore, for fast discharge EDLCs, imidazolium-based RTILs with short chains should be selected, largely because of the strong explosive power, and then charged before the second stage of discharge. For equipment that requires a slow and deep discharge, it is more appropriate to choose imidazolium-based RTILs with long chains because their two-stage connection is much smoother and has less energy leakage.

### 3.3. Whole Process of Discharging and Charging

**Figure 8:**
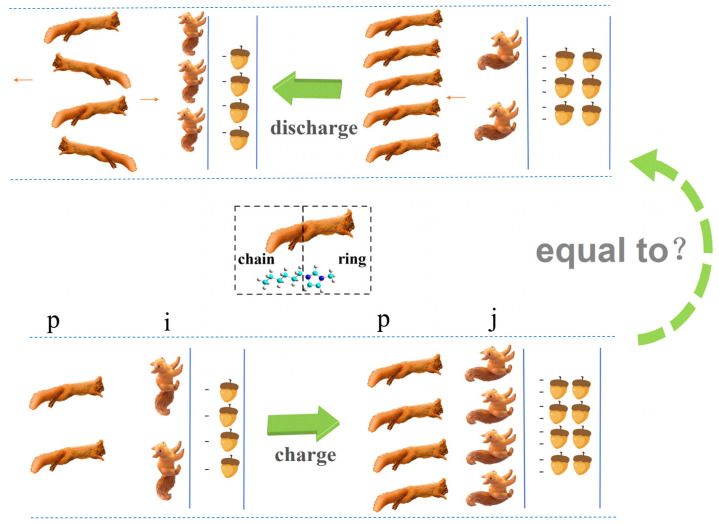
Schematic description of the charging and discharging cycle for imidazolium-based cations. The blue line represents the surface of the negative electrode. The number of nuts and squirrels show the trend of changing |σ| and cations, respectively. Blue dotted lines represent the periodic boundary conditions in the MD simulation. Brown arrows represent the directions of the moving cations.

Based on the discharging and charging process of imidazolium-based cations (which have longer alkyl chains) near the negative electrode, we can schematically depict this circle more vividly the use of the analogy of naughty squirrels. A larger |σ| in the electrode is represented by more nuts. In the beginning of the charging process in Figure 8, the number of nuts is not sufficient to attract more squirrels into the EDL. Therefore, there is sufficient room for them to lie their tails on this plane. Following this, an increasing amount of nuts attracts an increasing number of squirrels to the EDL, to the point where they have to stand up their tails, ensuring there is enough room for more companions to enter this layer. Furthermore, when the amount of nuts and squirrels match each other, there is sufficient room for all squirrels to have standing tails. Following that, other squirrels attracted by more nuts can only reside in the second layer, which is where overcrowding occurs.

Moreover, the discharging process begins, and a smaller amount of nuts leads to some squirrels leaving the compact layer for the crowding layers (the excess layers of counterions). An increasing number of squirrels move into the crowding layers, and the number in these layers is even larger than that in the compact layer during a certain stage of the discharging process shown in Figure 8. Finally, some squirrels move to the bulk liquid, yet some of them move in the opposite direction from the crowding layers to join squirrels in the compact layer once again.

Because of the two-stage discharge process of imidazolium-based ions [57], their discharging process is not equal to the reverse charging process. However, for other kinds of ions, such as surfactant-like ions [AOT]^−^ or NAIL ions [BF_4_]^−^, there is no two-stage discharging process, meaning their discharging process is nearly equal to the reverse charging process [57]. Therefore, the different ions should appear so.

## 4. Complex RTIL-Based Electrolyte Systems

### 4.1. RTIL–Solvent Mixtures

The relatively high concentration of cations and anions in neat RTIL electrolytes results in a strong van der Waals force and a coulomb ordered arrangement between the anions and cations. In comparison to neat RTILs, the majority of organic solvents have a larger electrochemical stability window, lower viscosity, greater conductivity, and a lower dielectric constant. As a result, diluting RTILs with organic solvents can weaken the van der Waals force, dismantle the Coulombic ordered arrangement between anions and cations, and make RTIL-based systems capable of having high conductivity, low viscosity, and low melting points, thus further lowering impedance and enhancing magnification performance. This process can also be used to regulate the working capacitance of RTIL-based electrolytes.

**Figure 9:**
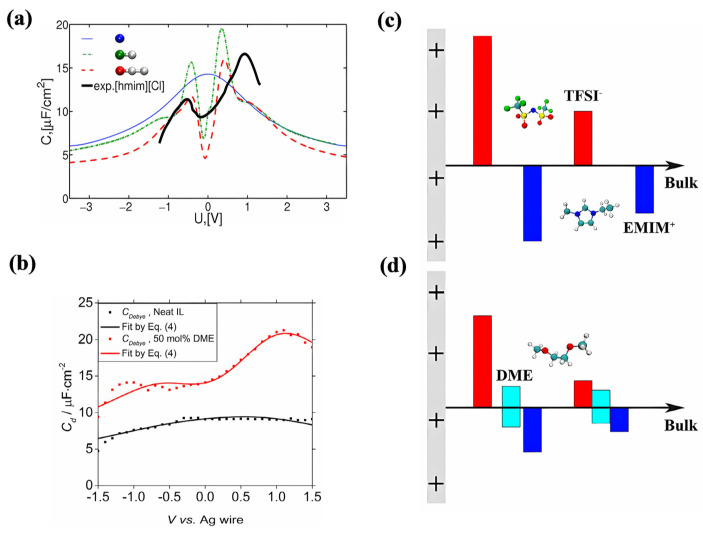
The influence of extending the neutral carbon chain and adding organic solvents to the structure of the EDL and energy storage. (**a**) Differential capacitance in the MD simulation and experiment for the electroneutral mixture of charged beads [34]. Copyright2010, Elsevier. (**b**) The capacitance of the neat RTIL and RTIL–solvent mixture electrolytes with 50% mol solvent [60]. Copyright2021, Elsevier. (**c**,**d**) Schematic drawings of the EDL structures of plain RTIL and RTIL–solvent mixtures close to an electrode surface that is positively charged [60]. Copyright2021, Elsevier. The red, blue and cyan bars represent anions, cations, and solvent, respectively.

In 2010, N. Georgi et al. [34] discovered, via MC simulations, that extending the neutral carbon chain of RTILs can change the capacitance shape from bell-shaped to camel-shaped, as shown in Figure 9a. If the neutral carbon chains were extended to a certain length, they would have a very effective energy storage performance at high temperatures (130~200 °C) [42]. Adding organic solvents to neat RTILs can also change the shape of the capacitance curve from bell-shaped to camel-shaped. According to Yan et al.’s research [60], the presence of organic solvents can transform the bell-shaped differential capacitance curve into a camel-shaped curve, thus considerably increasing the system’s capacitance, as shown in Figure 9b. This resembles increasing the nonpolar chains of neat RTILs, as shown in Figure 9c,d. The overscreening effect was suppressed by nonpolar domains that were formed by solvents or nonpolar chains.

Often, this curve may be another shape. Carolina Cruz et al. [61] investigated EDLs with RTIL–solvent mixtures that were on the verge of demixing via MD simulations. In addition to the well-known bell-shaped and camel-shaped capacitances, they showed the advent of a bird-shaped capacitance. The bird shape is between these two extremes, whereas the camel shape and bell shape emerge for electrodes that are ionophilic and ionophobic, respectively. This shows that the curve shape of capacitance may act as an electrochemical signature of the electrode’s affinity for ions.

**Figure 10:**
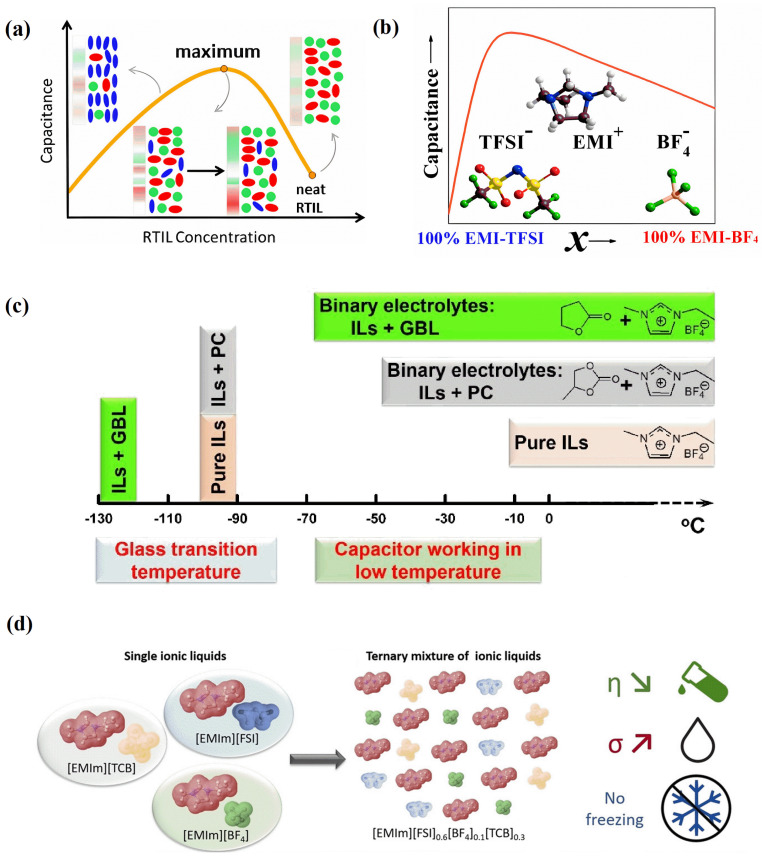
Concentration fluctuations and capacitive responses of electric double-layer capacitors with RTIL mixtures and RTIL–solvent mixtures. (**a**) Concentration changes and capacitive behavior in concentrated ionic solutions [66]. Copyright2016, American Chemical Society. (**b**) Concentration fluctuations and capacitive responses of EDLCs with RTIL mixtures [67]. Copyright2016, American Chemical Society. (**c**) [C_2_mim][BF_4_]-GBL binary electrolyte for a high-performance graphene-based capacitor operating at −70 °C and 3.7 V [65]. Copyright2018, Royal Society of Chemistry. (**d**) Neat RTILs mixed to formulate the ternary [C_2_mim][FSI]_0.6_[BF_4_]_0.1_[TCB]_0.3_ electrolyte with improved transport characteristics and a wide range of liquid states [68]. Copyright2021, Wiley.

Researchers have increased the power density by adding organic solvents to reduce the viscosity of the system while attempting to form nonpolar regions in the solvent in order to reduce the energy loss caused by the overscreening effect. L. J. A. Siqueira et al. [62] found that adding acetonitrile does not change the charge distribution on the electrode surface, but, within a suitable voltage range, can significantly reduce the viscosity of the RTIL-based electrolyte, thereby accelerating its charging speed and improving the power density. Ilhan A. Aksay et al. [63] added three different organic solvents, namely acetonitrile, propylene carbonate, and 1,2-dichloroethane, to the hydrophobic system [C_2_mim][NTf_2_]. It was found that the lowest value of differential capacitance would first rise and then fall as the concentration of RTILs increased, with its peak value rising from approximately 5 to 10% of the current ionic molar concentration, as shown in Figure 10a. This nonmonotonic change led to many studies focusing on underscreening, which pertains to the anomalously long electrostatic screening length that emerges when adding solvents.

Yan et al. [60] increased the content of acetonitrile in RTILs from 50% to 95%, and found that the shape of the differential capacitance curve changed from a bell-shape to a camel-shape. Moreover, increasing the degree of polarization of the electrode significantly increased the differential capacitance. In addition, the system with 50% ethylene glycol dimethyl ether (DME) and pure RTILs was compared.

Travis Douglas et al. [64] found that the crowding layer (excess counterion layer from the compact layer), which forms when pure [TDTHP][NTf_2_] is dissolved in strongly polar propylene carbonate (PC) or weakly polar dimethyl carbonate (DMC), is not a pure anion, but rather contains three to five times as many anions as cations and roughly the same amount of solvent as the bulk solution. The layer thickness and charge density at any given applied voltage both decrease as the dilution increases. This rule is particularly significant when neat RTILs in the RTIL–solvent mixture are greater than 50 vol%.

RTILs have a low ion conductivity, high viscosity, and a high melting point, resulting in poor low-temperature performance. Compounding other solvents with ionic liquids into binary electrolytes is the most commonly used technical approach, but it often significantly reduces the voltage window while expanding low-temperature performance, which contrasts the original intention of increasing the voltage window. Recently, Qian et al. [65] discovered a binary electrolyte, obtained by compounding the solvent γ-butyrolactone (GBL) with [C_2_mim][BF_4_]. The glass transition point for [C_2_mim][BF_4_] fell from −95 °C (for pure [C_2_mim][BF_4_]) to −126 °C (for the binary electrolyte), and the melting point (~15 °C) vanished. At −70 °C and 3.7 V, the binary electrolyte likewise had a high energy density of 61 W h kg^−1^.

### 4.2. RTIL Mixtures

Regulating electrolytes through RTIL mixtures mainly refers to mixing two RTILs with the same anion or cation, ensuring that cation (or anion) regulation can be performed without changing the anion (or cation). Similar to RTIL–solvent mixtures [66], RTIL mixtures also have a volcano-shaped curve for capacitance, with a change in proportion for one certain type of RTIL in the electrolyte [67], as shown in Figure 10b. On the other hand, RTIL mixtures can also make EDLCs work more effectively at low temperatures [68], as shown in Figure 10d.

Generally, EDLCs with carbon electrodes and organic electrolytes (especially based on low-viscosity acetonitrile) can operate effectively, even at low temperatures, unlike batteries, which experience a sharp decline in performance due to slow redox reaction rates. However, because the amount of charge stored in static electricity depends on the surface area of the EDL, the energy output of the EDL is lower than that of batteries. Based on the adjustable characteristics of RTIL electrolytes and carbon electrodes, François Béguin et al. [68] reported the excellent electrochemical performance of EDLCs at low temperatures (as low as −40 °C). The chemical formula of the ternary mixed RTIL electrolyte prepared in the article is [C_2_mim][FSI]_0.6_[BF_4_]_0.1_[TCB]_0.3_, as shown in Figure 10d. The ternary mixed electrolyte can remain in its liquid form at temperatures greater than −99 °C (−99 °C is its glass transition temperature). The ternary mixed RTIL electrolyte exhibits relatively low viscosity and high conductivity, with a viscosity η = 23.6 mPs at 20 °C and an ionic conductivity σ = 14.2 mS cm^−1^. The EDLCs exhibit excellent low-temperature energy storage performance, which can rival the traditional RTIL-based devices composed of organic electrolytes in this work.

However, the works on RTIL mixtures are limited to molecular simulations, and, currently, the main research work takes the form of experiments and classical density functional theory (cDFT). Mixing RTIL with another RTIL or solvents can alter their bulk properties, such as diffusion rate, viscosity, and conductivity. In addition, RTIL mixtures may have an excellent operating potential window (OPW) and long cycle performance. Using experiments and cDFT, the prediction can determine the optimal composition to maximize EDL capacitance, while having extended the OPW and enhanced the ion dynamics. For example, Lian et al. [67,69] investigated the potential window, interface structure, capacitance, and energy density of an RTIL mixture. Additionally, a volcano-shaped curve representing the theoretical results shows that capacitance varies nonlinearly with ion composition. The maximum capacitance of the [C_2_mim][NTf_2_]/[C_2_mim][BF_4_] mixture is approximately 4:1. Due to the presence of smaller [BF_4_]^−^ anions, the layered structure was significantly reduced, and the contact density of the counterions [C_2_mim]^+^ on the electrode surface increased. Molecular simulation has occasionally been employed to assist in explaining certain findings in experiments; for instance, Wang et al. [70] used MD simulations to observe details of RTIL mixtures, thus supporting their experimental observations. Currently, there is limited work from molecular simulation in the field of RTIL mixtures, indicating potential for further exploration in this direction in the future.

### 4.3. Water in RTIL Electrolytes

Water is a common impurity in RTILs, and most RTILs are hygroscopic. It is difficult to entirely remove the water from RTILs. Even hydrophobic ions have a high capacity for water absorption. The inevitable presence of water at the interfaces of RTILs and solid surfaces is problematic for energy storage. It is necessary to conduct studies on the adsorption of water at these interfaces, the impact on the molecular structure and dynamics of the interfaces, and any practical consequences [71].

This section will be divided by subheadings, and will provide a concise and precise description of the experimental results, their interpretation, as well as the experimental conclusions that can be drawn.

**Figure 11:**
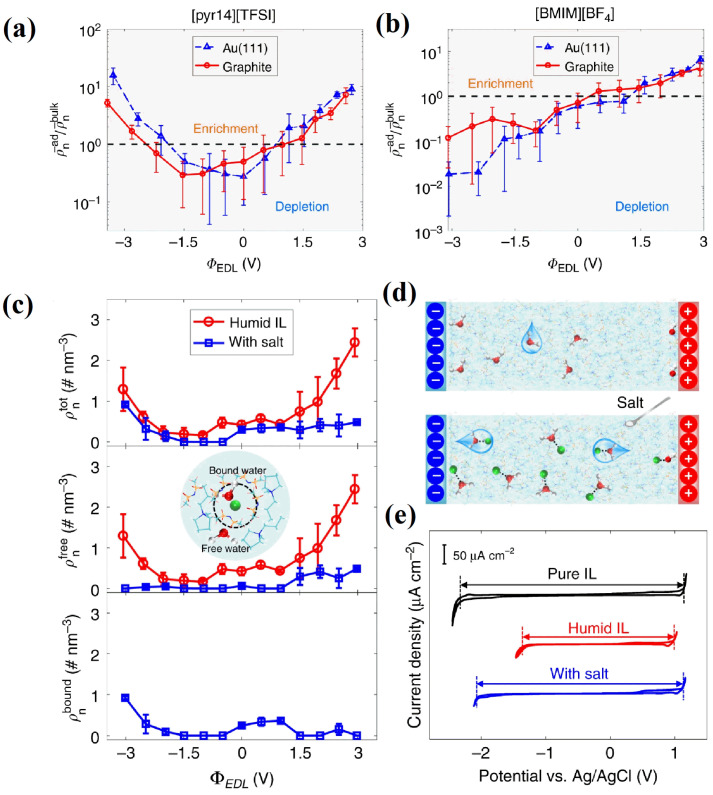
Water electrosorption for neat RTILs and the effect on the interfacial water from adding salt near electrodes. According to the electrical double-layer potential (ΦEDL), (**a**,**b**) exhibit water adsorption from the humid [pyr_14_][NTf_2_] and [C_4_mim][BF_4_], respectively [73]. Copyright2018, Springer Nature. (**c**) Salt or salt-free water electrosorption from humid [pyr_13_][NTf_2_]. The number densities of the total (*ρ*^tot^ _n_), free (*ρ*^free^ _n_) and Li^+^-bound water (*ρ*^bound^ _n_) in the interfacial area are shown in the top, middle, and bottom panels, respectively [74]. Copyright2020, Springer Nature. (**d**) A diagram showing how salt affects water electrosorption. (**e**) Electrochemical window of electrolytes [74]. Copyright2020, Springer Nature.

Qiao et al. [71] revealed the microscopic distribution of a small amount of water in RTILs at charged interfaces and its variation with the applied voltage on the graphene electrode. They elaborated on the formation and mechanism through the use of MD simulations. The imidazole ring parallel to the surface of the negative electrode can explain the larger amount of water enrichment near the positive electrode and, conversely, the lower amounts of water enrichment near the negative electrode. Using MD simulations, Zhang et al. [72] investigated the effects of various RTIL types and water molecule concentrations on the RTIL–mica interfaces, with a particular focus on the water distribution at mica surfaces. Additionally, they discovered that due to the respective RTIL hydrophobicity and ion size, more water collected at the interface with the hydrophobic [C_2_mim][NTf_2_] than in the case of the hydrophilic [C_2_mim][BF_4_]. As the research advanced, the water adsorption behavior was studied continually using complementary MD simulations and cyclic voltammetry (CV). Bi et al. [73] focused on the electrosorption behavior of water in RTILs on two different surfaces (gold and graphene) using two different RTILs (one hydrophilic and one hydrophobic), concluding that, in general, water will be depleted on negatively charged electrodes in hydrophilic RTIL systems, as shown in Figure 11a,b.

Meanwhile, for hydrophobic RTIL systems, Chen et al. [74] suggest a very simple idea, which consists of adding a Li ion-based salt, as shown in Figure 11e. The lithium “traps” the water molecules far from the interface, resulting in a substantial broadening of the electrochemical window. Experiments were performed to validate the idea, and MD simulations allowed us to understand these mechanisms. This has inspired a series of recent works concerning water-in-salt electrolytes and, more generally, studies where water acts as a reactant rather than a solvent.

## 5. The Influence of Novel Electrodes

### 5.1. Investigations of Porous Materials

**Figure 12:**
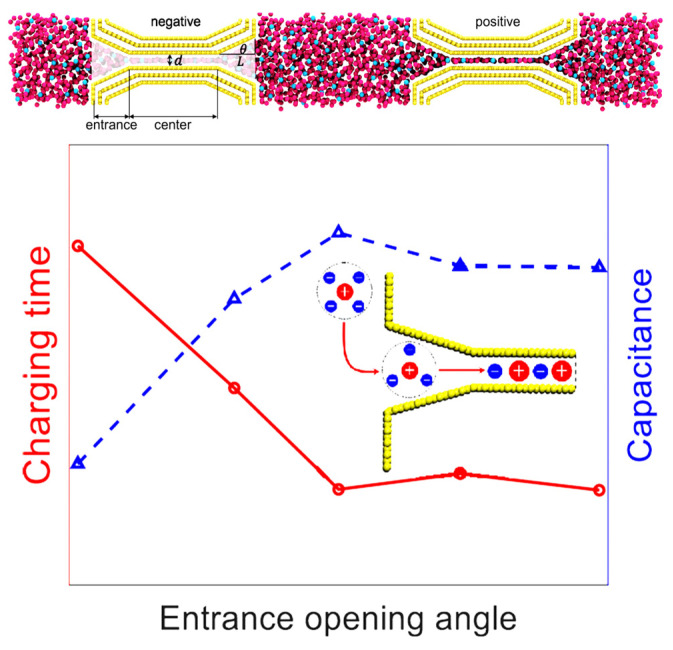
Capacitive behavior of a nanoporous supercapacitor with different opening angles and the schematics of simulation systems [76]. Copyright2023, American Chemical Society.

Porous carbons are currently the most widely used commercially available supercapacitor energy storage material. Due to the uneven distribution of pore sizes (from micropores to mesopores and even macropores), there is a relatively large amount of research on molecular simulations. Mo et al. [75] reported that, although ion diffusion is faster in negatively charged narrow pores, their charging speed is slower due to the phenomenon of the “overfilling” of ions during the charging process, thus making the ion desorption path more tortuous. Compared with single hole electrodes, the phenomenon of ion “overfilling” also occurs in porous electrodes, but the process of ion “defilling” in porous electrodes is accelerated. In addition, the charging of porous electrodes is accompanied by the phenomenon of “overcharging”, which will further accelerate the charging of porous electrodes. Furthermore, they can optimize the synergy between nanoporous carbons and RTILs, significantly enhancing the energy density of supercapacitors. Porous electrodes were also designed through the introduction of an optimized horn-like [76] entrance to the nanopore, as shown in Figure 12, which can concurrently improve supercapacitors’ charging dynamics and energy storage.

The reverse Monte Carlo method is suitable for constructing molecular simulation models for the pore size distribution and the pore structure of coconut shell activated carbon (CSAC). The results indicate that the nonuniform pore size distribution of CSAC electrodes, as well as their pore morphology, can effectively reduce the size effect caused by nanoconfined spaces, thus making the microscopic arrangement and dynamic transport of ions much more similar to the behavior in the extracellular electrolyte.

Based on the characteristics of metal organic frameworks (MOFs) that have molecular-scale models which can reflect actual structures, Bi et al. [77] used MD simulation calculations as the main research method, with experimental verification, to study the capacitance characteristics of a new type of supercapacitor, composed of conductive MOFs and RTILs. The MOF material with a metal organic structure forms the positive and negative electrodes of the supercapacitor, and the outer space of the positive and negative electrodes is filled with RTIL ions. After applying a fixed interface potential to the positive and negative electrodes, ions diffuse toward the outer surface of the electrode material and adsorb on it. The charges on the electrode material rearrange with the distribution of electrolyte ions at the interface. Upon overcoming the port energy barrier of nanopores, ions are adsorbed into the pores of MOF materials. Increasing the interface potential can enhance the adsorption of ions on the outer surface of the electrode material and within the pores. Overall, the influence of the interface potential on the electronic charge on the outer surface of the electrode material is much smaller than the net charge inside the pores. The net charge on the outer surface is ignored, and the surface charge density is determined using the electrolyte ion density distribution in the electrode material channel. The surface charge density increases monotonically with increasing interface potential, and the surface charge density is derived from the interface potential in order to obtain the corresponding differential capacitance. The evolution trend of capacitance in the positive and negative electrodes is different because the density distribution of anions and cations in the positive and negative electrodes is different. The differential capacitance is asymmetric relative to the zero potential point. When the interface potential is high, the energy density of the 0.81 nm channel is higher than that of both the 1.57 nm and 2.39 nm channels.

### 5.2. Investigations of Two-Dimensional and Other Low-Dimensional Nanomaterials

The positive and negative ions that make up RTILs are present in equal amounts. These liquids exhibit electrical neutrality in their entirety as a result of Coulombic ordering, in which alternating-charge ion shells develop around a center ion, which has gained the interest of many researchers. Mo et al. [78] studied the charging kinetics of RTILs in confined nanopores through the use of MD simulations. The charging kinetics of ions exhibit a nonmonotonic relationship between pore size and charging rate. Energy storage can be promoted in nanopores of 0.45 nm and 0.75 nm. Their work also further explained the mass transfer mechanism of the accelerated charging as follows: the transition state of the interlayer structure within the pore accelerates the charging of the nanopore. The one-layer ion distribution perpendicular to the aperture direction allows ions to freely transport in the direction perpendicular to the hole wall, thus reducing the impact of the wall surface on ion diffusion. The transition state of two-layer ions parallel to the pore wall reduces the activation energy of ion motion and accelerates ion diffusion. Therefore, RTILs confined in two-dimensional and other low-dimensional nanomaterials may demonstrate a unique performance in energy storage.

**Figure 13:**
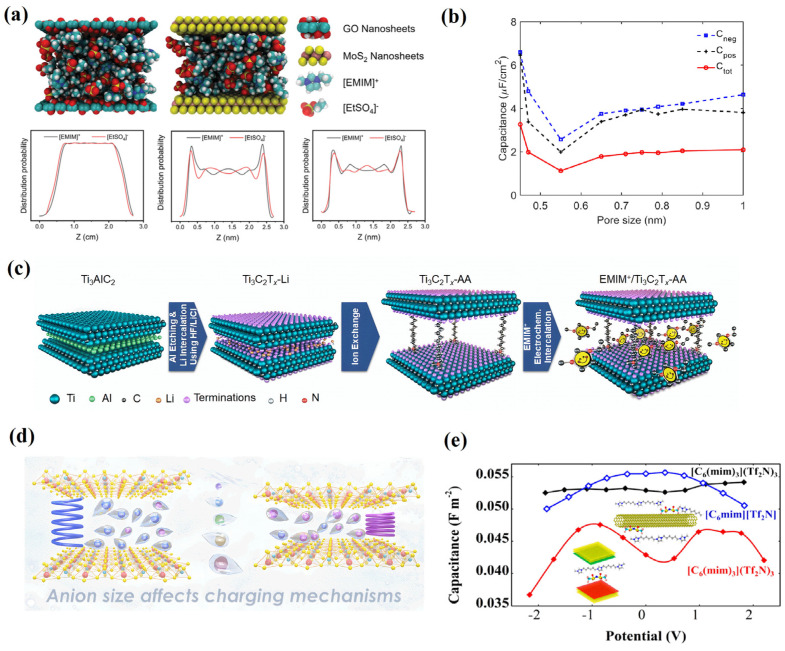
Electrodes of two-dimensional and other low-dimensional nanomaterials. (**a**) The structure and dynamic change of RTIL in graphene oxide (GO) and molybdenum disulfide (MoS_2_) [79]. Copyright2022, Wiley. (**b**) The relationship between the specific capacitance and pore size [78]. Copyright2020, American Chemical Society. (**c**) MXenes with various interlayer spacings achieved by using alkylammonium cations of various chain lengths [81]. Copyright2021, Wiley. (**d**) Volume growth or contraction in stacked electrode materials [82]. Copyright2020, Elsevier. (**e**) The adaptation of tricationic ILs (TILs) in the TIL/graphene system and CNT-based supercapacitors [83]. Copyright2023, American Chemical Society.

Dong et al. [79] used MD simulations to study the structure and dynamic change of RTIL in graphene oxide (GO) and molybdenum disulfide (MoS_2_). Unlike the random uniform distribution of pure RTIL, the specific distribution of [C_2_mim][EtSO_4_] in the width direction of the GO and MoS_2_ limits shows significant layered distribution characteristics, as shown in Figure 13a. The high-density layer of [C_2_mim][EtSO_4_] is located on both sides of the GO and MoS_2_ nanochannels, with two smaller density peaks located at the center of the GO and MoS_2_ nanochannels. The low density of [C_2_mim][EtSO_4_] in the centers of the GO and MoS_2_ nanochannels provides more separation regions for cations and anions, therefore promoting the dissociation of RTIL. In addition, these areas provide more space for RTIL transmission. The above results demonstrate the promotion effect of the two nanomaterials on the conductivity of the RTIL. Leonardo José Amaral Siqueira [80] also performed atomistic MDs to investigate the behavior of three different RTILs in contact with pores formed by GO electrodes. The oxygenated groups adsorbed on the surface of the GO lead to greater ionic mobility and a greater density of states, while maintaining the same specific area of pure graphene.

Xu et al. [82] focused on using MD simulations to investigate MXenes, a two-dimensional layered electrode material, and IL-based electrolytes. Their simulation results show that the diffusion characteristics of electrolyte ions in 0.7 nm layers are ~1.3–2 times higher than those of other layers (1.0 nm, 1.4 nm) and bulk in nanolimited environments. In addition, they observed periodic changes in the number of anions between the electrode layers, the structure of the double layer, and the arrangement of polar molecular dipoles during the dynamic charge-discharge cycle.

However, the latest results from experiments show that a high capacitance can provide a large specific capacitance, resulting in a high energy density with an interlayer spacing of approximately 2.2 nm. This conclusion seems to conflict with the interlayer spacing of 0.7 nm uncovered by the MD simulations above. However, the MD simulations only investigated an interlayer spacing of 0 to 1 nm, as shown in Figure 13b, and showed a trend of increasing slowly during the interlayer spacing of 0.7 to 1 nm, deriving from the results of Mo et al. [78]. Experimental results were obtained by Liang et al. [81]. As a supercapacitor electrode, MXene can obtain ultrahigh capacitance in aqueous electrolytes; however, its energy density is constrained by the electrolyte’s constrained potential window. The operating potential window offered by organic electrolytes and RTILs is larger (2.5 to 4 V). However, the large size of IL cations, which are much larger than conventional hydrogen protons or metal cations, prevents them from embedding into the interlayer of the MXene electrode, thus resulting in a capacitance much lower than that of aqueous electrolytes. Therefore, Liang et al. [81] prepared MXenes with various interlayer spacings using alkylammonium cations of various chain lengths in order to study the impact of the layer spacing on the electrochemical performance of MXenes, as shown in Figure 13c. MD simulations were also utilized to elucidate the structure of ions inside the pores. The interlayer spacing of 2.2 nm provided the greatest performance among the preintercalated MXenes investigated, compared to the spacings of 1.3, 1.4, 1.5, 1.6, and 2.5 nm.

Xu et al. [82] employed MD simulations and continued to obtain significant information related to MXenes in RTIL-based electrolytes. By tracking the number and rearrangement of ions in the electrode holes, they obtained a quantitative description of the various charge storage methods in both the negative and positive electrodes [84]. Heterogeneous charge ion implantation is the method largely used to achieve charge storage in the negative electrode (69% to 94%, varying with the variety of surface functional groups), while in the positive electrode hole, this is mainly realized through the co-ion/counterion exchange process (65% to 88%). This asymmetric energy storage mechanism results in an increase in the total number of ions in the negative electrode and a decrease in the total number of ions in the positive electrode during charging. The variation patterns of the ions in the two electrodes are concurrent with the dynamic volume change trend of the electrode observed in the experiments. Researchers reported a thorough MD simulation study that closely aligned with the experimental results regarding the impact of the nature of functional groups and anion size on charging mechanisms and volume expansion/contraction in layered materials utilized as electrodes for energy storage applications, as shown in Figure 13d.

Carbon nanotubes (CNTs) are typical one-dimensional tubular carbon nanomaterials. Molecular dynamic simulations of carbon nanotubes show that their inner and outer surfaces exhibit different energy storage mechanisms. For the outer surface, due to the influence of their curved surface, the differential capacitance of carbon nanotubes does not change with the electrode potential. For the inner surface, due to the confined space, ions may undergo “exclusive internal solvation”, exhibiting significant nanoscale size effects.

Tricationic ILs (TILs) [85,86] in CNT-based supercapacitors were shown to be adaptable through the use of MD modeling, according to Li et al. [83]. As the CNT curvature rises, the EDL capacitance in the TIL grows dramatically, and the capacitance of the TIL/CNT systems is larger than the capacitance of the TIL/graphene systems. The results showed the good performance of the one-dimensional electrode material. Furthermore, it is confirmed that the TIL can store more energy at high potentials than monocationic IL. This indicates that the improvement of EDLCs can be multifaceted, not only in electrolytes, but in electrode materials as well.

### 5.3. Controllable External Potential Conditions

There are two common methods for the MD simulation of capacitors; these are the constant charge method (CCM) [87,88] and the constant potential method (CPM). The CPM can respond well to the interface charge polarization effect, which is more consistent with the actual process than the CCM (based on a uniform and constant charge distribution on the electrode surface). Merlet et al. [89] noted that these two methods match well at ideal plane potentials; however, at complex interface potentials, the CPM predicts a better interfacial electrolyte density distribution than the CCM. This is because the accumulation of interfacial electrolytes can form stronger interfacial electrostatic interactions. Additionally, the CPM is favored because it has self-consistent electrode charge adjustments, which the CCM lacks, and can result in proper charging dynamics and heat production.

The galvanostatic mode, in which an electrode is applied with a constant electric current, has been widely used in practical applications [90], as well as fundamental electrochemical studies (such as galvanostatic charge-discharge, or GCD) [91]. They are, however, unable to provide information regarding the charging kinetics in this mode. As a result, the majority of current GCD simulations are based on the CCM, with each electrode atom having its equivalent charge set and changing linearly over time (this approach is referred to as GCD-CCM in the following). However, the precision of GCD-CCM, particularly for nanoporous systems, is yet unknown since there is no self-consistent adjustment of electrode charges.

In 2021, Zeng et al. [92] developed a molecular simulation method with equal potential to simulate the constant current charging and discharging process of supercapacitors (this method is called GCD-CPM in the following). The results show that, for supercapacitors with nanoporous electrodes, the GCD-CPM can not only obtain a charge and discharge process that is consistent with the experiment for RTIL-based electrolytes, but can also describe the kinetic hysteresis phenomenon of ion adsorption and desorption during the charge and discharge process on the molecular scale, explaining its formation mechanism also. Therefore, the GCD-CPM can more accurately simulate the charging and discharging processes of supercapacitors. This method can be used for the molecular simulation of EDLCs and will become mainstream in the future.

## 6. Conclusions and Outlook

Molecular simulations are a powerful tool for the study of the behavior and performance of EDLCs at the atomic level. By examining the interactions between ions and ion pathways, these simulations can provide insights into the distribution and movement of electrolyte ions, which are key factors in energy storage and dynamic behavior. These insights can be used to enhance the electrocapacitive performance of EDLCs made from nanomaterials. Molecular simulations also enable researchers to optimize the structure and composition of EDLCs, thus improving their capacitance and energy density, and to develop new electrode materials and electrolytes that can enhance their performance.

Additionally, experimental methods are constantly evolving. For instance, substantial developments in liquid phase AFM have made the detection of vertical layer separations possible, and can even create 3D density maps of EDLs utilizing sensitive force measurements and EC-3D-AFM [41]. Furthermore, cutting-edge CP-3D-AFM was further developed [37], which could observe the true spatial charge distribution on the electrode surface and EDL at an angstrom resolution. In addition, some new experimental technologies have been developed, such as the simple open circuit potential method [36]. With the progress of experimental technology, the detection technology of RTIL-based electrolytes in EDLCs could become increasingly clear, meaning more microscopic phenomena will be revealed in the future.

**Figure 14:**
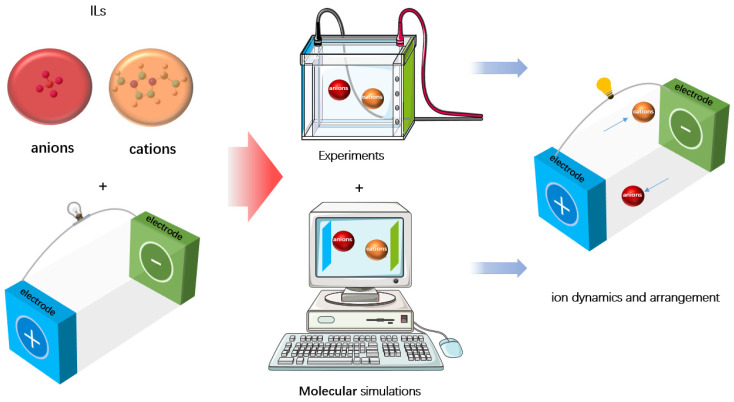
The relationship between experiments and molecular simulations, reflecting on their application in RTIL-based EDLCs.

With the advancement of experimental technology, these effects have been verified by experiments. The simulation prediction of the capacitance curve, which was bell-shaped or camel-shaped, coincides with the experiment for pure RTILs. For complex systems, such as RTIL–solvent mixtures and RTIL mixture systems, both molecular simulations and experiments have reported that the change in capacitance curves follows a volcano-shaped trend with RTIL concentration. Finally, experiments and molecular simulations have maintained synchronous development in recent years, as shown in Figure 14, and this paper discussed their relationship, reflecting on their application.

However, there are still some challenges in this field that require further investigation, as follows: (a) Currently, the studies focusing on molecular simulations and RTIL mixtures are insufficient, and this kind of investigation will reveal more physical mechanisms for EDLCs with RTIL mixtures. (b) Predictions based on molecular simulations are verified by experiments, while others are not. For instance, many studies have obtained experimental results related to the overscreening effect, but the obtained results on the crowding effect have been insufficient; furthermore, there are several disagreements regarding the crowding effect. A future direction might be to intensify the crowding effect in the experimental setting within the OPW of RTILs. (c) According to the predictions of molecular simulations and theoretical studies, the crowding effect appears to be harmful to energy storage. However, in real testing, certain findings indicate that crowding can actually improve capacitance, thus defying many predictions. Future studies should focus more on this topic. (d) Researchers have recently concentrated on the suppression of the overscreening effect from self-assembly behavior near the electrodes. Self-assembly behavior occurs when the length of the carbon chain reaches a specific point. According to Perkins et al.’s research [49,50], rather than a tendency toward a gradual transition, there is no self-assembly phenomenon that varies with the length of the carbon chain until the carbon chain length reaches a particular point. Self-assembly phenomena can also differ in certain ways as a result of different charged heads. These changes in self-assembly behavior require more detailed exploration in future studies. 

## Data Availability

The data presented in this study are available in article.
